# Addressing the mental health needs of children affected by HIV in Rwanda: validation of a rapid depression screening tool for children 7–14 years old

**DOI:** 10.1186/s12887-020-02475-1

**Published:** 2021-01-29

**Authors:** Agnes Binagwaho, Eric Remera, Alice Uwase Bayingana, Darius Gishoma, Kirstin Woody Scott, Madeline Goosman, Eliza Campbell, Mawuena Agbonyitor, Yvonne Kayiteshonga, Sabin Nsanzimana

**Affiliations:** 1 University of Global Health Equity, Kigali, Rwanda; 2 Harvard Medical School, Boston, MA USA; 3 Dartmouth College Geisel School of Medicine, Hanover, NH USA; 4 Rwanda Biomedical Center, Kigali, Rwanda; 5 University of Rwanda College of Medicine and Health Sciences, Kigali, Rwanda; 6 University of Michigan, Ann Arbor, Michigan USA; 7 University of Virginia, Charlottesville, VA USA; 8 Partners in Health, Freetown, Sierra Leone

**Keywords:** Depression scale, Validation, Child mental health, HIV/AIDS, Rwanda

## Abstract

**Background:**

Depression in children presents a significant health burden to society and often co-exists with chronic illnesses, such as human immunodeficiency virus (HIV). Research has demonstrated that 10–37% of children and adolescents living with HIV also suffer from depression. Low-and-middle income countries (LMICs) shoulder a disproportionate burden of HIV among other health challenges, but reliable estimates of co-morbid depression are lacking in these settings. Prior studies in Rwanda, a LMIC of 12 million people in East Africa, found that 25% of children living with HIV met criteria for depression. Though depression may negatively affect adherence to HIV treatment among children and adolescents, most LMICs fail to routinely screen children for mental health problems due to a shortage of trained health care providers. While some screening tools exist, they can be costly to implement in resource-constrained settings and are often lacking a contextual appropriateness.

**Methods:**

Relying on international guidelines for diagnosing depression, Rwandan health experts developed a freely available, open-access Child Depression Screening Tool (CDST). To validate this tool in Rwanda, a sample of 296 children with a known diagnosis of HIV between ages 7–14 years were recruited as study participants. In addition to completing the CDST, all participants were evaluated by a mental health professional using a structured clinical interview. The validity of the CDST was assessed in terms of sensitivity, specificity, and a receiver operating characteristic (ROC) curve.

**Results:**

This analysis found that depression continues to be a co-morbid condition among children living with HIV in Rwanda. For identifying these at-risk children, the CDST had a sensitivity of 88.1% and specificity of 96.5% in identifying risk for depression among children living with HIV at a cutoff score of 6 points. This corresponded with an area under the ROC curve of 92.3%.

**Conclusions:**

This study provides evidence that the CDST is a valid tool for screening depression among children affected by HIV in a resource-constrained setting. As an open-access and freely available tool in LMICs, the CDST can allow any health practitioner to identify children at risk of depression and refer them in a timely manner to more specialized mental health services. Future work can show if and how this tool has the potential to be useful in screening depression in children suffering from other chronic illnesses.

## Background

Depression is a serious co-morbid condition associated with a variety of chronic medical illnesses, often limiting a patient’s ability to achieve optimal outcomes [1, 2]. According to the World Health Organization (WHO), the prevalence of depression is estimated to be 4.4% [3]. The burden is even higher among individuals suffering from human immunodeficiency virus (HIV), ranging from 10 to 37% [4, 5]. Children with HIV are uniquely vulnerable to depression relative to their counterparts [6]. This added burden of depression to that of HIV is concerning as it negatively impacts quality of life and adherence to HIV treatment [4].

Though challenges exist, there is effective treatment for depression in patients with HIV [3, 4]. However, low-and-middle income countries (LMICs) face chronic shortages of mental health providers; fewer than 25% of affected individuals ever access mental health treatment [7, 8]. In response, the WHO launched the mental health Gap Action Programme (mhGAP) in 2008, https://apps.who.int/iris/handle/10665/43809, to support initiatives that would aid in more rapid screening, diagnosis, and treatment of mental illness, including depression, in LMICs [9, 10]. Rwanda, a country of 12 million people in Sub-Saharan Africa, embraced the principles of mhGAP but barriers persist in meeting its ideals of providing adequate, timely mental health care to all in need. For instance, a 2018 national survey showed that 86% of Rwandans who met criteria for a mental disorder did not seek care [9]. When studying the prevalence of depression among children affected by HIV [11, 12], it was found that 25% of children living with HIV in Rwanda also met criteria for depression [11].

The burden of depression among children living with HIV, especially in LMICs where access to mental health services is limited, underscores the need for increased attention to the psychological needs and well-being of this vulnerable group. In response to these findings, Rwanda’s Ministry of Health recommended routine screening for depression among children living with HIV. Though well-intentioned, there were no easily accessible tools to Rwandan clinicians or the few mental health providers that existed in the country. As such, screening for depression among children rarely occurred [13]. Therefore, the purpose of this study is to describe both the development of Rwanda’s Children Depression Screening Tool (CDST) as well as the process of validating this tool by comparing results with a Rwandan national survey. The CDST was designed to be a practical resource for Rwandan clinicians to rapidly and effectively screen children living with HIV in the country.

## Methods

### Creation of the CDST

Though validated screening tools have been helpful with optimizing health outcomes in depression [14, 15], there have been relatively few efforts to develop and validate screening tools for depression among the pediatric population with HIV in LMICs. A literature review of the existing validated depression assessment tools available around the world, and specifically in Rwanda, both in terms of their content, availability, ease of use, predictive validity, and cultural sensitivity, was conducted in 2015, finding that the tools available at the time were either insufficient or unaffordable in the Rwandan context. There were no other rapid assessment tools for depression previously validated in Rwanda other than the Children’s Depression Inventory (CDI), which is a tool available only by cost for each single use. Given the prohibitive cost to expand this nationally in a LMIC, this motivated the development of a new freely-accessible alternative that would allow Rwanda to screen children for depression nationally.

With the support of experienced Rwandan psychologists, psychiatrists, and pediatricians, the CDST was developed and copyrighted as a free, open source tool. The development of the CDST was based on a variety of existing international tools for diagnosing depression, including the WHO and Diagnostic Statistical Manual of Mental Disorders, fourth edition (DSM-IV). In addition, the CDST was designed to be validated against a national standard interview guide in Kinyarwanda, which was created based on WHO norms to standardize interviews for diagnosing depression among children in the country. For instance, the Rwandan expert team also considered previous studies on local concepts of depression-like problems in Rwandan children and adolescents affected by HIV/AIDS. Majority of expression used such as *agahinda kenshi* (persistent sorrow) and *kwiheba* (severe hopelessness) were found to correlate with some of the DSM-IV criteria [16, 17]. These culture-specific expressions serve as examples for how the CDST took into account the Rwandan context when designing this screening tool. The CDST was designed to be a practical, usable tool for non-mental health specialists such as nurses and general practitioners working in primary health care settings.

The CDST is composed of 11 questions relating to the themes of mood, interest in leisure, hope of living, fatigue, psychomotor activity, sleep, appetite, concentration, interpersonal relationships, suicidal thoughts or suicide attempts and the feeling of guilt. Each question had a four point answer scale (0 = absence of symptoms, 1 = symptom sometimes present, 2 = symptom frequently present, 3 = symptom always present) to assess the mood, interest in leisure, hope of living, fatigue, psychomotor activity, sleep, appetite, concentration, interpersonal relationships, suicidal thoughts or suicide attempts and the feelings of guilt. The CDST was translated by Rwandan psychologists and psychiatrists into Kinyarwanda, ensuring that local culture was taken in account, and was subsequently back translated to English by a different team of psychologists and psychiatrists. The tool was beta tested in two randomly selected sites (one urban and one rural) to identify ambiguities and assure question clarity. Based on the feedback received from users and respondents, the tool was updated into a final version used for data collection.

### Validation of the CDST

#### Study design and sampling

The CDST validation study was carried out from August 1, 2019 to November 5, 2019. The population of interest were children living with HIV in Rwanda. The study was limited to children between the ages of 7 to 14 as it required that children have a known positive HIV status and ability to participate in the depression screening. According to the national HIV program, there were approximately 5000 children [18] aged 7 to 14 years living with HIV on antiretroviral therapy (ART) in Rwanda; it was expected that approximately 25% would meet criteria for depression. This population along with the tool parameters (7% absolute precision, design effect of 1.2, and adjusting for the non-response rate and the expected specificity of 90%), a final sample size of 283 was needed for this analysis. According to guidelines on HIV disclosure among children in Rwanda, it is recommended to provide complete disclosure of HIV/AIDS status before 14 years. Previous study indicated that children aged 7 and above in Rwanda were more likely to be aware of their HIV status than their younger counterparts [19]. In order to select the study sites and participants, a list of health facilities per province offering HIV care and treatment services to children was extracted from Rwanda’s Ministry of Health Management Information System. To assure adequate representation from rural areas, two urban health facilities and three rural health facilities were randomly selected as study sites from each of Rwanda’s five provinces. In each of the 25 selected facilities, a study assistant randomly selected 12 participants who would undergo both the CDST screening tool as well as a diagnostic interview by mental health professionals, the latter of which was considered the gold standard for diagnosing depression.

### Standardized clinical interview for DSM-IV (SCID)

All participants who underwent the CDST screening tool were also interviewed by one of five mental health professionals. These professionals had 5 to 20 years of clinical experience and had a systematic interview tool to assess participants as to whether or not they met criteria for Major Depressive Disorders from the DSM-IV. The Structured Clinical Interview for DSM- IV (SCID) is widely considered the gold standard in both clinical practice and research [20]. The procedure has been validated in East African settings [21] and the Kinyarwanda Structured Clinical Interview for Depression have been widely used in different studies in the Rwandan context [11, 22]. Children and their guardians were asked to participate in the study on a voluntary basis and understood that they could end their participation at any moment with no consequences to their right to access health services. All children suspected to have mental health problems were referred for appropriate care and follow up regardless of their participation in the study.

#### Study variables

Beyond the data captured from the CDST and interview process, the following participant demographics, socioeconomic, and clinical status variables were captured for the purposes of the study, including: age, gender, education, survivorship of parents, person living with the child, the person who disclosed the HIV status to the child, time on ART treatment, and recent HIV viral load.

### Statistical analysis

The results of the CDST were compared to those of the SCID gold standard interview (as conducted by the trained senior mental health professionals). This permitted for the calculation of the sensitivity, specificity, positive predictive value, and negative predictive value estimates for the CDST. For every sampled child, a CDST score was calculated, then at every cut-off score, a Receiver Operating Characteristic (ROC) analysis was performed to determine the recommended cut-off that would make this an appropriate screening tool for depression among Rwanda’s population.

## Results

### Validation study participants and demographics

The CDST was finalized in May 2019. A total of 296 children (49% male and 51% female) participated in the study with ages ranging from 7 to 14 years (mean: 12, SD: 1.9). The majority of children had both parents alive (93.6%) and were living with their parents (86.1%), while 13.9% lived with their siblings, relatives or another guardian. The majority (94.2%) attended primary school and 5.8% attended secondary school. In terms of HIV disclosure prior to the study, 53.3% of respondents found out their HIV status from their parents/guardians while the remaining 42.1% had their status disclosed by the assistance of a health care provider with or without parental involvement. All children were receiving ART, with the majority of children (80%) showing a viral load below the threshold of < 1000 RNA copies/ml. Non-suppressed HIV viral load was found in 19% of the participants. With regards to geography, children living in the Western province had higher rates of depression relative to those in other areas. [Table 1].

**Table 1:** Demographic Characteristics and prevalence of depression

	N	%	Prevalence of depression	
			%	95% CI
	296		**14.2**	[9.6,20.4]
**Province**				
East	60	20.3	11.7	[4.5,27.1]
West	70	23.6	24.3	[17.0,33.4]
City of Kigali	57	19.3	15.8	[6.3,34.2]
North	53	17.9	3.8	[1.2,11.1]
South	56	18.9	12.5	[5.4,26.4]
**Age of the child (years)**				
9-Jul	46	15.5	6.5	[2.2,17.9]
12-Oct	114	38.5	9.6	[5.1,17.5]
13–14	136	45.9	20.6	[13.8,29.6]
**Sex of the child**				
Male	145	49	11	[6.5,18.0]
Female	151	51	17.2	[11.3,25.3]
**Residence facility**				
Urban	146	49.3	13.7	[7.5,23.8]
Rural	150	50.7	14.7	[9.0,23.1]
**Current education level**				
Primary	275	94.2	14.2	[9.8,20.1]
Secondary	17	5.8	17.6	[4.9,47.0]
**Parents are alive?**				
Both Alive	277	93.6	14.1	[9.5,20.4]
One Alive	19	6.4	15.8	[3.8,46.8]
**Person living with the child when out of school**				
Parents	255	86.1	13.3	[8.2,20.9]
Siblings/relatives/Guardian	41	13.9	19.5	[10.8,32.7]
**Time on ART**				
Below 24 months	27	9.1	14.8	[6.8,29.4]
25–60 months	61	20.6	13.1	[6.1,25.9]
61–120 months	139	47	12.9	[8.0,20.2]
121+ months	69	23.3	17.4	[7.8,34.3]
**Who disclosed the HIV Status?**				
Parents/ Guardian	152	53.3	13.8	[9.1,20.3]
Parent with Health Care Providers	120	42.1	15.8	[9.3,25.8]
Health Care provider	13	4.6	7.7	[0.9,44.4]
**Recent viral load suppressed (< 1000 copies)**				
No	50	19.2	16	[7.7,30.3]
Yes	211	80.8	12.3	[8.3,18.0]

### Prevalence of depression using clinical interview/gold standard

According to the SCID gold standard process, a total of 14.2% (95% CI: 9.6, 20.4) of children were found to have depression. Depression was highest among adolescents aged 13–14 years relative to younger children (*p* < 0.05). Further the province of residence was associated with depression, with a higher prevalence in the western province (p < 0.05). [Table 1].

### Identifying depression using the CDST tool

The CDST scores were compared to the results of the clinical interviews to determine sensitivity (proportion of children who have depression according to clinical interview and who are correctly identified by CDST) and specificity (proportion of children without depression and who have been correctly identified as non-depressed by the CDST). The optimal cut-off point for an accurate determination of the risk of depression (a positive screen) using the CDST tool was determined. A threshold score of 4 was found to be associated with 95.2% of children with depression diagnosis, while a 10 cut-off score was associated only with 40.5% of the subjects with depression. Analyzing the CDST at different scores, the specificity was 86.6% at the cut-off point of 4 and 99.6% at the cut-off point of 10. The cut-off points of 5 to 6 appeared to provide the optimal levels of sensitivity and specificity compared to the rest of the scores. [Table 2].

**Table 2:** Sensitivity and specificity by depression scale

Scale Cutoff	Sensitivity	Specificity	Area under the curve (AUC)	Positive predictive value	Negative predictive value
Four	95.2%	86.6%	0.909	54.1%	99.1%
Five	92.9%	91.3%	0.921	63.9%	98.7%
**Six**	**88.1%**	**96.5%**	**0.923**	**80.4%**	**98.0%**
Seven	78.6%	98.0%	0.883	86.8%	96.5%
Eight	73.8%	99.2%	0.865	93.9%	95.8%
Nine	57.1%	99.6%	0.784	96.0%	93.4%
Ten	40.5%	99.6%	0.700	94.4%	91.0%

**Fig. 1:**
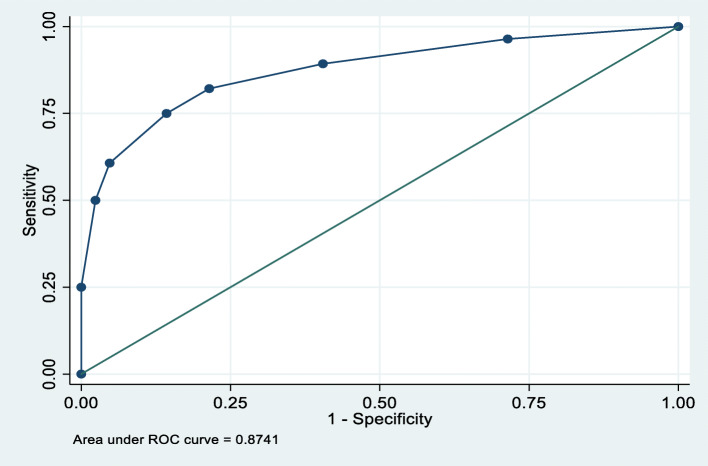
Receiver Operating Characteristics (ROC) Curve of CDST Tool

Finally, a ROC analysis was completed to determine the global functioning of the scale as well as overall accuracy of the CDST tool in identifying depression risk. The cut-off point of 6 achieved the greatest area under the curve (92.3%) with a marked increase in specificity. [Fig. 1].

## Discussion

This article summarizes the creation of a newly developed, freely-accessible tool in Rwanda for screening depression among children suffering from HIV, how it was adapted to the Rwandan context, and how the CDST tool was validated.

This analysis found that 14% of children living with HIV in Rwanda screened positive for depressive symptoms. Though not a majority, this suggests the importance of having readily accessible tools in Rwanda to identify this uniquely at-risk population so that both their HIV burden and mental health suffering can be minimized. Similar to prior work, this study showed that children living with HIV ages 7–14 years in Rwanda had the highest prevalence of depression in the western province [11]. Several factors may contribute to this pattern including, but not limited to, geographical accessibility; the region is mountainous with limited access to existing primary health facility network [23], socioeconomic factors; the highest rates of extreme poverty and malnutrition can be found in the south and west of the country [24], and other historical reasons such as the traumatic impact of the genocide [25].

Compared to prior studies that utilized the CDI tool, this study found a lower prevalence of depression among this vulnerable population (14% with the CDST versus 25% with the CDI) [11]. Though lower, this rate still falls within the range of prior studies suggesting that depression rates among children with HIV varies from 10 to 37% [4]. This discrepancy between the tools may also be contextual; the CDI tool was created for children in the USA, while the CDST was adapted to the Rwandan local setting, which may alter its screening accuracy.

Results from this study indicated that the presence of depressive symptoms was also associated with being female and an adolescent (ages 13–14). Studies conducted in other settings have shown no differences in depression rates by sex in childhood and prepubescence; however, a gap appears to emerge between the ages of 11 and 15 as girls become approximately twice as likely as boys to experience depression [26–29]. This gender gap appears to persist through adulthood where depression affects women more than men [26–30]. A variety of deeply rooted social factors may contribute to the gender-linked vulnerability to depressive symptoms not only in Rwanda but also in other contexts, which are explored elsewhere and merit further investigation for addressing.

Prior studies have shown higher rates of depression and behavioral health challenges among adolescent boys and girls with perinatally acquired HIV, which are associated with decreased medication adherence and increased transmission risk [30, 31]. This may be partly explained by the transition to puberty, adolescent difficulties, parental-child relationship, and circumstances surrounding HIV-status disclosure. During the recruitment phase of this study, it was observed that disclosure of the diagnosis of HIV to children remained a challenge for parents and healthcare providers. According to national guidelines, HIV positive status should be carefully disclosed to affected individuals starting by age 7, however, more than half of children aged 7–9 considered for recruitment were unaware of their HIV status and were thus excluded in this study. Children had learned of their HIV status during the pre-adolescence or adolescence period; this may have led to added emotional distress among those aged 13–15.

The results of this study indicated that the CDST was found to be feasible tool to implement in Rwanda and that it was comparable to the gold standard SCID. When designing a screening tool, it is important to optimize the benefit of not missing any affected children while also not burdening the health system (i.e. using a tool with a high false-positive rate) [32]. This analysis showed the process for identifying an optimal cut-off score for the CDST (6 points). As such, this study suggests that the CDST serves as a practical, quality screening tool for identifying children with HIV at risk for depression and thus more timely referrals to mental health specialists. Further, using this score, this sets a limit on the false-positive rate in order to conserve resources for the few available mental health professionals in Rwanda. This will help to improve access to appropriate mental health prevention, treatment, and supportive services for children in Rwanda at risk of suffering from depression.

### Limitations

Though the CDST offers a unique, context-specific tool for screening depression, there are important limitations to this study. First, this is limited to a single cohort within a single country, therefore the generalizability to other LMIC settings is unclear. Secondly, since the CDST was tested and validated only among children living with HIV among a particular age group [7 to 14], it is not possible to confirm the validity of it beyond this cohort. Nonetheless, given the importance to Rwanda for identifying children at risk of depression, the intent of the CDST is to be available for all children across all chronic diseases. This will require further efforts to expand the validation population for the tool. Additional next steps include adopting the tool in the Rwandan national program and continuously monitor its benefits in improving mental health outcomes. Further, the CDST tool is freely available for other LMICs to adapt and use, thereby trying to address the important financial barriers that previously hindered these resource-limited settings from national screening efforts among this vulnerable population.

## Conclusion

Currently, the majority of children living with HIV in LMICs are not systematically screened for depressive symptoms. These barriers make it difficult for children in these settings to receive timely mental health care and treatment, which has a negative impact on their overall long-term health outcomes. This study shows how a newly-developed, freely-accessible screening tool created in a LMIC setting was validated for accurately screening children living with HIV for depression. This tool was designed with the intent that it can be used by general practitioners, nurses, and midwives in their clinical practice to improve assessment, referral and treatment of depression among children and adolescents living with HIV to the limited mental health practitioners who live in LMIC settings. Implementing this valid, freely available assessment tool has the potential to strengthen the mental health referral process, creating a path to the development of an effective child mental health strategy in Rwanda and beyond.

## Data Availability

The dataset analysed during the current study is available from the corresponding author on reasonable request.

## Abbreviations

ART: Antiretroviral therapy
ARVs: antiretroviral medications
AUC: Area under the ROC curve
CDI: Children’s Depression Inventory
CDST: Child Depression Screening Tool
DSM-IV: Diagnostic and Statistical Manual for Mental Disorders, version IV
HIV: Human Immunodeficiency Virus
LMICs: Low- and Middle- Income Countries
ROC: Receiver Operating Characteristic
SCID: Standardized Clinical Interview for DSM-IV
WHO: World Health Organization
